# Oral administration of resveratrol reduces oxidative stress generated in the hippocampus of Wistar rats in response to consumption of ethanol

**DOI:** 10.3389/fnbeh.2023.1304006

**Published:** 2024-01-11

**Authors:** Addí Rhode Navarro-Cruz, Daniel Juárez-Serrano, Ivan Cesar-Arteaga, Ashuin Kammar-García, Jorge Alberto Guevara-Díaz, Obdulia Vera-López, Martin Lazcano-Hernández, Ivonne Pérez-Xochipa, Orietta Segura-Badilla

**Affiliations:** ^1^Departamento de Bioquímica-Alimentos, Facultad de Ciencias Químicas, Benemérita Universidad Autónoma de Puebla, Puebla, Mexico; ^2^Dirección de Investigación, Instituto Nacional de Geriatría, Mexico City, Mexico; ^3^Facultad de Medicina, Universidad Autónoma de Sinaloa, Culiacán, Mexico; ^4^Departamento de Nutrición y Salud Pública, Facultad de Ciencias de la Salud y de los Alimentos, Universidad del Bío-Bío, Chillán, Chile

**Keywords:** oxidative stress, hippocampus, ethanol, reactive oxygen species, resveratrol

## Abstract

**Introduction:**

Chronic ethanol intake has been found to favor hippocampal deterioration and alter neuronal morphological maturation; resveratrol has been suggested as an antioxidant that may counteract these effects. The objective of this study was to analyze the effect of resveratrol on oxidative stress markers, endogenous antioxidant system in the hippocampus, and the behavior of male Wistar rats administered different concentrations of ethanol.

**Methods:**

The animals, at 3 months old, were randomly distributed into 11 study groups (*n* = 6/group), orally administered (5 days on, 2 days off) with water (control), ethanol (10, 20, 30, 40 or 50%), or ethanol (10, 20, 30, 40 or 50%) plus resveratrol (10 mg/Kg/day) for 2 months. Subsequently, the production of nitrites, malondialdehyde, and 4-hydroxy-alkenal (HNE) and the enzymatic activity of catalase and superoxide dismutase (SOD) were quantified.

**Results:**

The levels of nitric oxide and lipid peroxidation products were significantly increased in each ethanol concentration and were statistically different compared to the control group; however, resveratrol significantly reduced oxidative stress caused by high ethanol concentration. The SOD and CAT did not present significant changes with respect to the controls in any of the study groups. In the different concentrations of ethanol used, GR increases significantly in the groups administered with resveratrol but not GPx. Resveratrol was shown to maintain the results similar to the control at most ethanol concentrations.

**Discussion:**

Our results suggest that resveratrol prevents oxidative stress induced by ethanol in the hippocampus by decreasing cellular lipid peroxidation, but does not prevent the activation of catalase or SOD enzymes; however, allows glutathione to be kept active and in adequate concentrations in its reduced form and avoids alterations in the locomotor system.

## 1 Introduction

Ethanol is a psychoactive substance with dependence-causing properties, and it has been widely used in many cultures for centuries. Alcohol abuse causes serious social and economic problems, as well as various pathological consequences, representing more than 5.1% worldwide (WHO, 2022). Alcohol exerts negative effects on different organs, considerably affecting the liver (Jeon and Carr, 2020), heart (Roerecke, 2021), skeletal muscle (Laudato et al., 2021), pancreas (Petersen, 2021), and brain (Topiwala and Ebmeier, 2017; Lees et al., 2020).

In this sense, it is known that ethanol abuse induces detrimental effects in the central nervous system, one of them being neuronal damage associated with increased oxidative-nitrosative stress and activation of the inflammatory cascade that ultimately results in structural and functional deficits of an organism (Tiwari and Chopra, 2013). In the brain, chronic alcohol consumption induces neuronal death by apoptosis in brain regions related to cognition, such as the hippocampus and the cerebral cortex of adult rats (Díaz et al., 2016), thus showing affectation of motor and cognitive functions, especially those related to fine motor behavior, attention, learning, and declarative memory.

Post-mortem studies have shown that alcoholic patients present a marked degeneration of the hippocampus characterized by a decrease in cell survival and alteration of cell morphological maturation (Golub et al., 2015; Zahr and Pfefferbaum, 2017). Increased oxidative stress, that emerges when an imbalance exists between reactive oxygen and nitrogen species formation and the capability of cells to clear them (Pizzino et al., 2017), causes damage to macromolecules and consequently increases loss of brain tissue, disorganization of the hippocampal detrital structure, and reduced levels of neurotrophins such as brain-derived neurotrophic factor (BDNF), neurotrophin-3 (NT-3), nerve growth factor (NGF), and glial cell-derived neurotrophic factor (GDNF) (Davis, 2014; Silva-Peña et al., 2018).

In recent years, it has been shown that a therapeutic option for the neurodegenerative effects caused by chronic ethanol intake is antioxidant therapy. In nature, there are several compounds with high antioxidant capacity, one of the most important groups is polyphenols, whose molecular structure is characterized by the presence of one or several phenolic rings (Costa et al., 2021). One of the most important polyphenols is resveratrol (3,5,4-trihydroxystilbene), naturally present in many plants and fruits in the diet, for example, grapes, blueberries, raspberries, blackberries, and products such as red wine (Snopek et al., 2018; Buljeta et al., 2023). Various properties of resveratrol are currently known including being antioxidant, anti-aging, cardioprotective, anticancer, anti-inflammatory, and chemoprotective, and possessing neuroprotective molecules (Baur and Sinclair, 2006; Flores et al., 2016).

In various stress models, resveratrol has been shown to have the ability to reduce the presence of free radicals such as reactive oxygen and nitrogen species (RNOS), decrease neuronal death, and strengthen the endogenous enzymatic antioxidant system. Within the first line of endogenous antioxidant defense, catalase and superoxide dismutase (SOD) are included, which participate in a chain preventing the formation of free radicals at the cellular level and rapidly neutralizing any molecule with the potential to become a free radical (Ighodaro and Akinloye, 2018).

*In vitro* and *in vivo* studies have shown that treatment with exogenous dietary antioxidants prevents neuronal apoptosis and avoids ethanol-induced spatial memory deficits (Sheth et al., 2009; Ataie et al., 2016). However, there are no reports showing the effect of resveratrol on the progression of oxidative stress and the state of the endogenous antioxidant system in the face of excessive consumption of ethanol. The objective of this study was to analyze the effect of resveratrol on oxidative stress markers, on the endogenous antioxidant system in the hippocampus, and on the behavior of male Wistar rats administered different concentrations of ethanol.

## 2 Materials and methods

### 2.1 Experimental design

In this experimental study, we used 66 male Wistar rats, 3 months old, weighing 280–320 g, obtained from the vivarium “Claude Bernard” of the Benemérita Universidad Autónoma de Puebla. The animals were kept under standard conditions with dark–light cycles of 12 h at a temperature of 21°C with *ad libitum* access to water and food.

Through random allocation (simple randomization), 11 study groups were formed (6 rats each), which were orally administered through an intragastric cannula with water (control), ethanol (10, 20, 30, 40, and 50%) or ethanol (10, 20, 30, 40, and 50%) plus resveratrol (10 mg/kg/day), for 2 months.

Ethanol (*d* = 0.789 g/mL) solutions were prepared with distilled water at concentrations of 10, 20, 30, 40, and 50%, which is equivalent, respectively, to doses of 78.9, 157.8, 236.7, 315.6, or 394.5 mg/kg. Ethanol was administered (1 mL/kg/day) for 5 days (Monday to Friday) with 2 days off (Saturday and Sunday) pattern (between 8:00 a.m. and 10:00 a.m.), to minimize the chances of gastrointestinal damage. The different concentrations of ethanol were prepared to model different types of drinks. In the ethanol + resveratrol groups, resveratrol (ResVitále®, *Polygonum cuspidatum*, USA) was diluted and administered together with ethanol, and the concentration of resveratrol was 5 mg/mL.

At the end of the follow-up, the rats were euthanized using ketamine/xylazine solution; to prepare the ketamine/xylazine solution, 4.0 mL of ketamine HCL (100 mg/mL) and 2.0 mL of xylazine (20 mg/mL) were mixed with 2 mL of sterile saline in a sterile vial. The ketamine/xylazine solution was administered intraperitoneally at a dose of 100 mg/Kg of ketamine and 10 mg/Kg of xylazine. The hippocampus was dissected bilaterally and stored at −70°C (Ultrafreezer716 Thermo Forma, Dreieich, Alemania) for the determination of oxidative stress markers. All procedures were approved by the Institutional Committee for the Care and Use of Laboratory Animals with project folio 100,426,322-CICUALVIEP-18/3 and were carried out according to the Official Mexican Standard NOM-62-ZOO-1999, as well as the “Guide for the Care and Use of Laboratory Animals” from Mexico. All procedures were carried out minimizing the unnecessary suffering of the animals. The study is reported in accordance with the Animal Research: Reporting of *In Vivo* Experiments (ARRIVE) guidelines.

### 2.2 Biochemical analysis

#### 2.2.1 Total protein quantification

Total proteins (TP) were quantified using Sedmak and Grossberg’s method, in which bovine serum albumin is used as a standard. Proteins were quantified in 2 μL of the supernatant plus 500 μL of the color reagent (0.06% Coomassie blue at 465 nm), brought to 1 mL with distilled water. The reaction product was read in a spectrophotometer (Lambda EZ 150, PerkinElmer, USA) at 620 nm. Protein concentration was determined by interpolating the optical density of the samples to the bovine serum albumin standard curve (1–10 μg) (Sedmak and Grossberg, 1977).

#### 2.2.2 Nitrite (NO_2_^−^) quantification

Nitric oxide (NO) production was analyzed using the NO_2_^−^ content in the supernatant obtained, utilizing the method of Tsikas, based on the Griess reaction. In this assay, the colorimetric reaction is induced by adding 100 μL of supernatant +100 μL of Griess reagent and 800 μL of H_2_O. Subsequently, the reaction product was read in a spectrophotometer at 540 nm. The NO_2_^−^ concentration was determined by interpolating the optical density of the samples on the NaNO_2_ standard curve determined for the assay (1–10 μg). The results were expressed as micromoles of nitrite per milligram of total protein (NO_2_^−^μM/mg TP) (Tsikas, 2007).

#### 2.2.3 Determination of MDA+ 4-HDA

The content of malondialdehyde (MDA) and 4-hydroxyalkenals (4-HDA) was tested using Erdelmeier’s method. Lipid peroxidation products were analyzed, using *N*-methyl-2-phenyl-indole as chromogenic reagent [10.3 mM]. For this, 100 μL of distilled water +100 μL of supernatant +650 μL of diluted solution I were added, shaken vigorously, and finally, added with 150 μL of methanesulfonic acid (MDA + 4-HDA determination) or 35% HCl (determination of MDA). The tubes were incubated at 45°C for 60 min (MDA) and 45°C for 40 min (MDA + 4-HDA), allowed to cool at room temperature for 5 min, and centrifuged for 15 min at 3,000 rpm. Subsequently, the absorbance of the reaction product was read at 586 nm. The concentration of MDA and 4-HDA was determined by interpolating the optical density of the samples on a standard curve of 1, 1, 3, 3, tetramethoxypropane (1–10 μg). The results were expressed as micromoles of MDA and 4-HDA per milligram of total protein (MDA or 4-HDA μM/mg TP) (Erdelmeier et al., 1998).

### 2.3 Enzymatic analysis

#### 2.3.1 Catalase enzyme activity

Catalase activity was determined spectrophotometrically by the method of Aebi; 635 μL of phosphate buffer [50 mM] pH 7.4, 330 μL of H_2_O_2_ [30 mM], and 35 μL of hippocampus supernatant were added to a quartz cell. The reaction was followed for 2 min, reading in minutes 0 and 2 at a temperature of 20°C, and the readings were made at 240 nm. To determine the enzymatic activity, the ΔOD was multiplied by the molar extinction coefficient (Aebi, 1984).

#### 2.3.2 Superoxide dismutase activity

The SOD activity was determined according to the method of Marklund and Marklund. The blank test of the uninhibited reaction was carried out until obtaining a ΔOD 0.020 ± 0.001. The enzymatic activity was calculated by means of the equation given below:


$$\left[\left(\frac{\text{Mean}\;\text{value}\;\text{of}\;\Delta OD\;\text{of}\;\text{the}\;\text{sample}\times 100}{\text{Mean}\;\text{value}\;\text{of}\;\text{the}\;\Delta OD\;\text{of}\;\text{the}\;\text{blank}}\right)-100\right]\times 0.6$$

Generally, 1 U of SOD is considered as the amount of enzyme that inhibits the pyrogallol autoxidation by 50% at 25°C and pH 8.2. The SOD activity is expressed as U/mg TP/min (Marklund and Marklund, 1974).

### 2.4 Glutathione system

The activity was determined according to the method of Juarez et al. (2023).

#### 2.4.1 Total glutathione (Total-GSH), reduced glutathione (GSH), and oxidized glutathione (GSSG)

The total content of GSH and GSSG was determined using the enzymatic recycling technique based on the use of the enzyme glutathione reductase (Juarez et al., 2023). Glutathione is oxidized by 5,5′-5-nitrobenzoic acid (DNTB) and reduced by nicotinamide adenine dinucleotide phosphate (NADPH) in the presence of glutathione reductase. To determine the concentration of total glutathione, the formation of 2-nitro-5-thiobenzoic acid was monitored at 412 nm, and the glutathione present was evaluated by comparing the result with the standard curve. When 2-vinylpyridine or 4-vinylpyridine (which does not inhibit glutathione reductase, GR) is used to mask GSH, the procedure is specific for GSSG.

#### 2.4.2 Determination of glutathione S-transferase enzymatic activity

The tissue was homogenized in 50 mM phosphate buffer; subsequently, the samples were centrifuged at 10,000 rpm for 10 min at 4°C. In total, 30 μL of the supernatant obtained previously was taken, and 125 μL of reagent 1, 15 μL of reagent 2, and 3.5 μL of reagent 3 were added. The DABS/min was obtained by monitoring the reaction for 5 min at 37°C at a length of 340 nm. One unit of glutathione S-transferase (GST) is defined as μmol of GSH-conjugated CDNB per minute, and the specific activity is represented as nanomoles per milligram of protein.

#### 2.4.3 Determination of the enzymatic activity of glutathione reductase

– Step A: 150 μL of the main reagent +30 μL of the regulatory buffer +10 μL of sample + 10 μL of 315 μM FAD were added. DABS/min was determined for 3 min in a spectrophotometer tempered at 340 nm (A).
– Step B: 20 μL of regulatory buffer +30 μL of GSSG were added. DABS/min was determined for 3 min in a spectrophotometer at 37°C at a length of 340 nm (B).

One GR unit is defined as micromoles of GSSG reduced per minute, and the specific activity is represented as nanomoles per milligram protein.

#### 2.4.4 Determination of the enzymatic activity of glutathione peroxidase

For the reaction, 125 μL of sample and 200 μL of coupling reagent were taken. The reaction contained 2 mM GSH, 0.15 U/mL GR, 0.4 mM sodium azide, 10 mM tert-butyl hydroperoxide, 0.5 mM NADPH, and 26 μL of sample (0.08–0.16 mg protein). One unit of glutathione peroxidase (GPx) was defined as micromoles of NADPH consumed per minute, and the specific activity is represented as nanomoles per minute per milligram (nM/min/mg) of protein.

### 2.5 Locomotor behavior and memory

#### 2.5.1 Open field

After the different treatments, the animals were tested for spontaneous locomotor activity in a new environment. Assays were performed as previously described (Hernández-Hernández et al., 2018). Locomotor activity was monitored in a wooden cubic device measuring 60 cm long × 60 cm wide and 60 cm high where the base is divided into three quadrants of 20 cm × 20 cm per side. The test consisted of placing the animal in the central quadrant and evaluating horizontal behavior (distance traveled), recording with a camera for 5 min. All animals were evaluated from 8:00 to 10:00 a.m.

#### 2.5.2 Novel object recognition test (NORT)

The method described by Pulido et al. (2019) was used. This is a behavioral assay commonly used for the investigation of various aspects of learning and memory in rodents. This test can be completed in 3 days: habituation (motor activity) day, short-term memory/training day, and long-term memory analysis day. Since rodents have an innate preference for novelty, if the animal recognizes the familiar object, it will spend most of its time on the novel object, in both tests. Due to this innate preference, there is no need for positive or negative reinforcement or long training programs.

##### 2.5.2.1 Phase 1: habituation day

The animals were habituated; for this purpose, the rat was placed inside the box for 5 min for exploration without objects; they were later placed again in an acrylic cage. Between each test, the area and the objects between each tested animal were thoroughly cleaned using 70% ethanol. This habituation is important for the animal to become familiar with the environment.

##### 2.5.2.2 Phase 2a: training phase

Twenty-four hours after habituation, the training phase was performed. The animal was placed equidistant from two identical objects and allowed to explore for 5 min. Between each test, the area and the objects between each tested animal were thoroughly cleaned using 70% ethanol.

##### 2.5.2.3 Phase 2b: short-term memory evaluation phase

After 2 h of the training phase, the short-term memory was assessed, using one of the objects used in the previous phase and a novel object in the work area. The objects were placed in the same location used in the previous phase. Free exploration was allowed for 5 min, and at the end, the area and the objects were thoroughly cleaned between each tested animal using 70% ethanol.

##### 2.5.2.4 Phase 3: long-term memory evaluation phase

After 24 h of the short-term memory assessment, one of the objects with which short-term memory was tested was replaced with a novel object, and free exploration was allowed for 5 min and thoroughly cleaned at the end. The area and the objects between each animal were tested using 70% ethanol.

During each stage, the time the animal touched the object with the nose and/or front paws or sniffed it was measured, and the recognition memory index was calculated using the following formula:


$$\text{Recognition}\;\text{memory}\;\text{index}=\frac{\text{NO}}{FO+\text{NO}}$$

where FO is the time with a familiar object, and NO is the time with a novel object.

### 2.6 Statistical analysis

Data are presented as mean ± standard error (SE). To determine the effect of resveratrol on markers of oxidative stress and endogenous antioxidants, a two-way fixed effects ANOVA model was used; to determine the differences between groups, pairwise comparisons were applied using Tukey’s *post-hoc* test, and the results were summarized as mean difference and 95% confidence interval. A value of *p* of <0.05 was considered a statistically significant difference. All analyses and graphs were performed in GraphPad Prism v.9.0.1 software.

## 3 Results

### 3.1 Effect of resveratrol on ethanol-induced changes in nitrite levels

**Figure 1:**
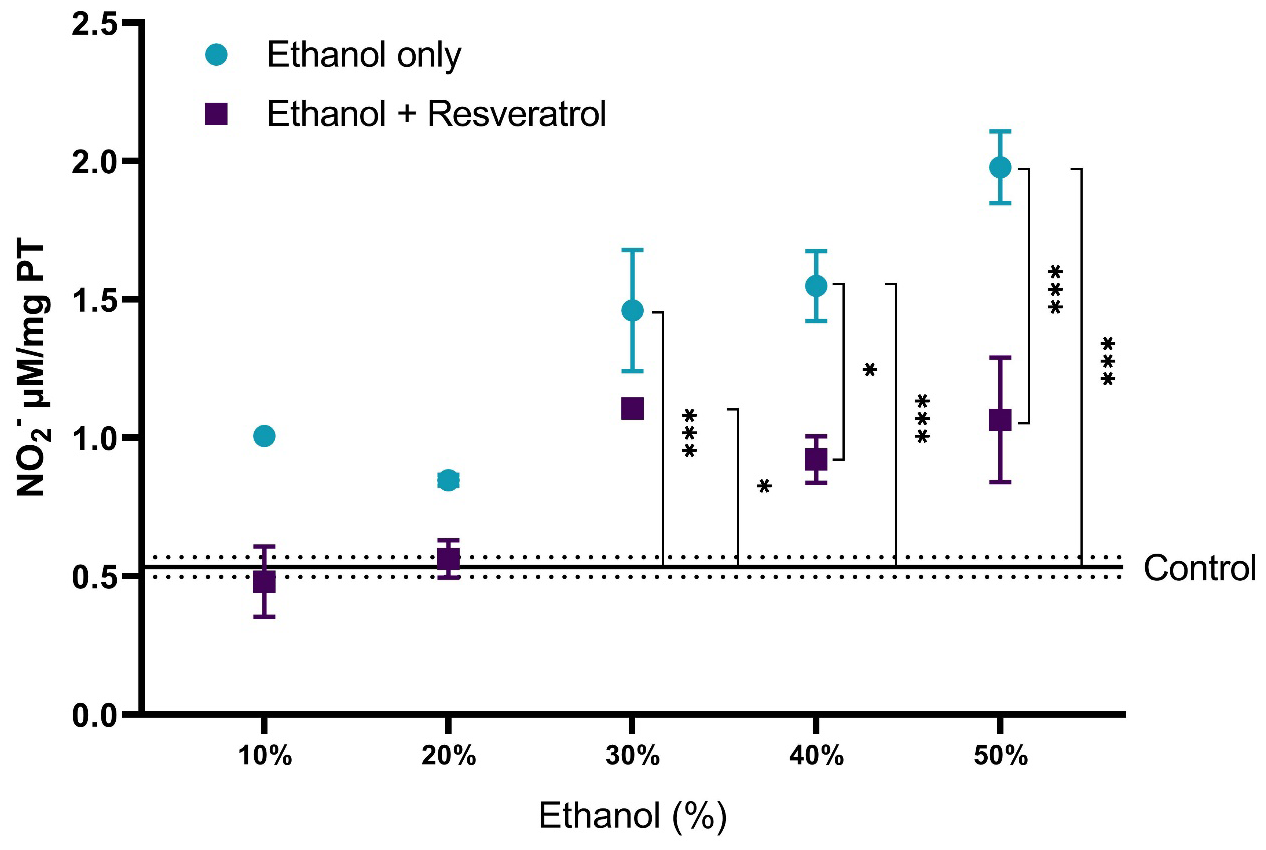
Antioxidant activity of resveratrol on nitrite production in the hippocampus of rats administered with ethanol. Data are presented as mean ± SE. The straight line is the mean of the control group, and the dotted line is the SE. Comparison is made using a two-way fixed effects ANOVA model, and *post-hoc* comparison was made using Tukey’s test. *p < 0.05, **p < 0.01, ***p < 0.001.

The results of the comparisons of nitrite production between the study groups are shown in Figure 1. The control group had a mean of 0.53 ± 0.036 NO_2_^−^ μM/mg TP. At ethanol concentrations of 10 and 20%, the ethanol only and ethanol + resveratrol groups had similar results to those observed in the control group. From the 30% concentration, the ethanol group showed higher levels of nitrites than the control group (30% MD = –0.92, 95%CI, −1.48 to −0.38, *p* < 0.0001; 40% MD = –1.01, 95%CI: −1.57 to −0.47, *p* < 0.0001 and 50% MD = −1.44, 95%CI: −1.99 to −0.89, *p* < 0.0001). The resveratrol group had similar results to the control group in all concentrations, with the exception of the 30% concentration (MD = –0.57, 95%CI: −1.21 to −0.02, *p* = 0.04). Between the ethanol and resveratrol groups, the ethanol-only group presented higher values of nitrite than the resveratrol group, but statistically significant differences were only observed at concentrations at 40% (MD = 0.63, 95%CI: 0.08 to 1.78, *p* = 0.01) and 50% (MD = 0.91, 95%CI: 0.36 to 1.46, *p* < 0.0001).

### 3.2 Effect of resveratrol on ethanol-induced changes in lipid peroxidation

**Figure 2:**
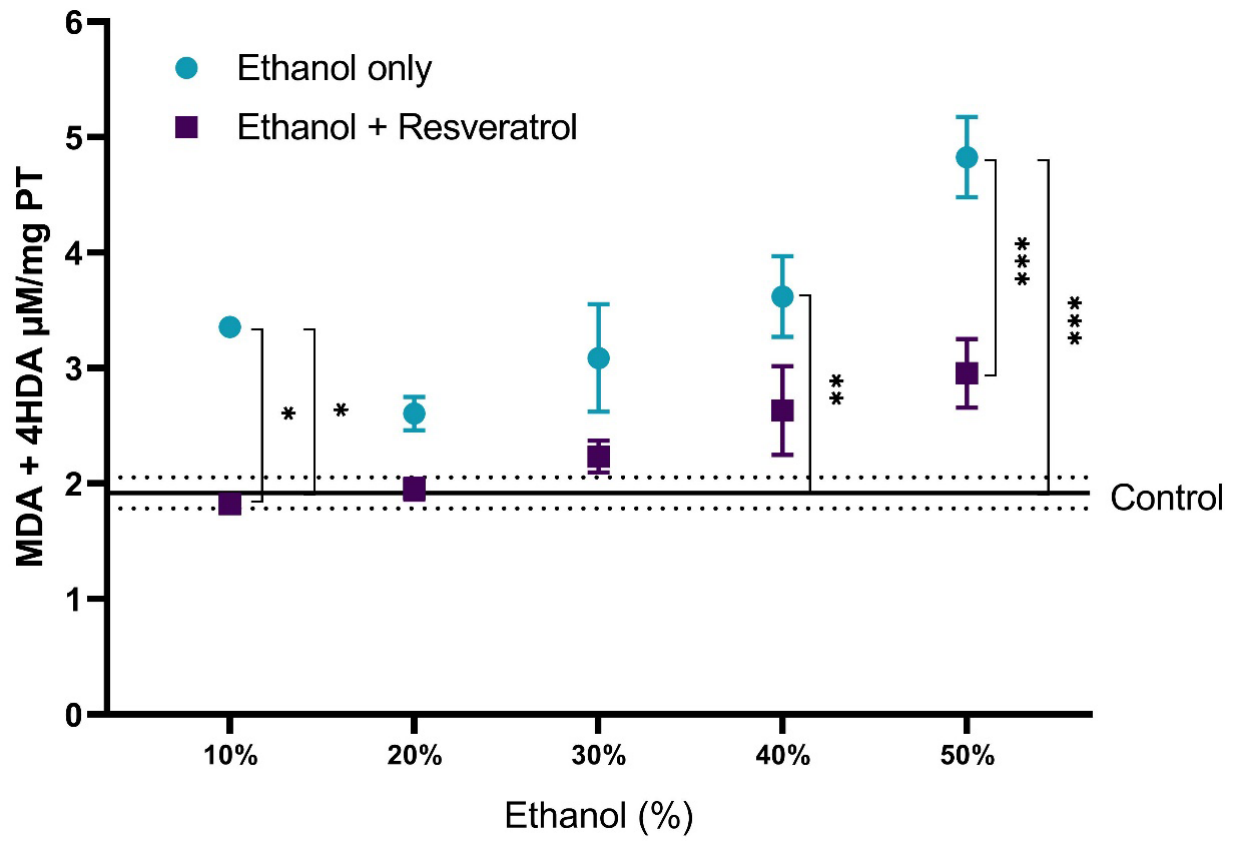
Antioxidant activity of resveratrol on lipid peroxidation products (MDA + 4-HDA) in the hippocampus of rats administered with ethanol. Data are presented as mean ± SE. The straight line is the mean of the control group, and the dotted line is the SE. Comparison is made using a two-way fixed effects ANOVA model, and *post-hoc* comparison was made using Tukey’s test. *p < 0.05, **p < 0.01, ***p < 0.001.

For the evaluation of lipid peroxidation products, the comparisons of MDA + 4-I between the study groups are shown in Figure 2. The mean of the control group was 1.92 ± 0.13 μM/mg TP, and it was observed that, in all ethanol concentrations, the resveratrol group did not have different values from the control (all *p* > 0.05), but the ethanol group was statistically higher than the control group at the concentrations of 10% (MD = –1.43, 95%CI: −2.67 to −0.20, *p* = 0.01), 40% (*p* = 0.002), and 50% (MD = 1.87, 95%CI: 0.64 to 3.11 *p* = 0.0004). Regarding the comparison between the ethanol and resveratrol groups, only differences were observed in the ethanol concentration at 10% (MD = 1.53, 95%CI: 0.29 to 2.75, *p* = 0.005) and 50% (MD = 1.87, 95%CI: 0.64 to 3.11, *p* = 0.0004).

**Figure 3:**
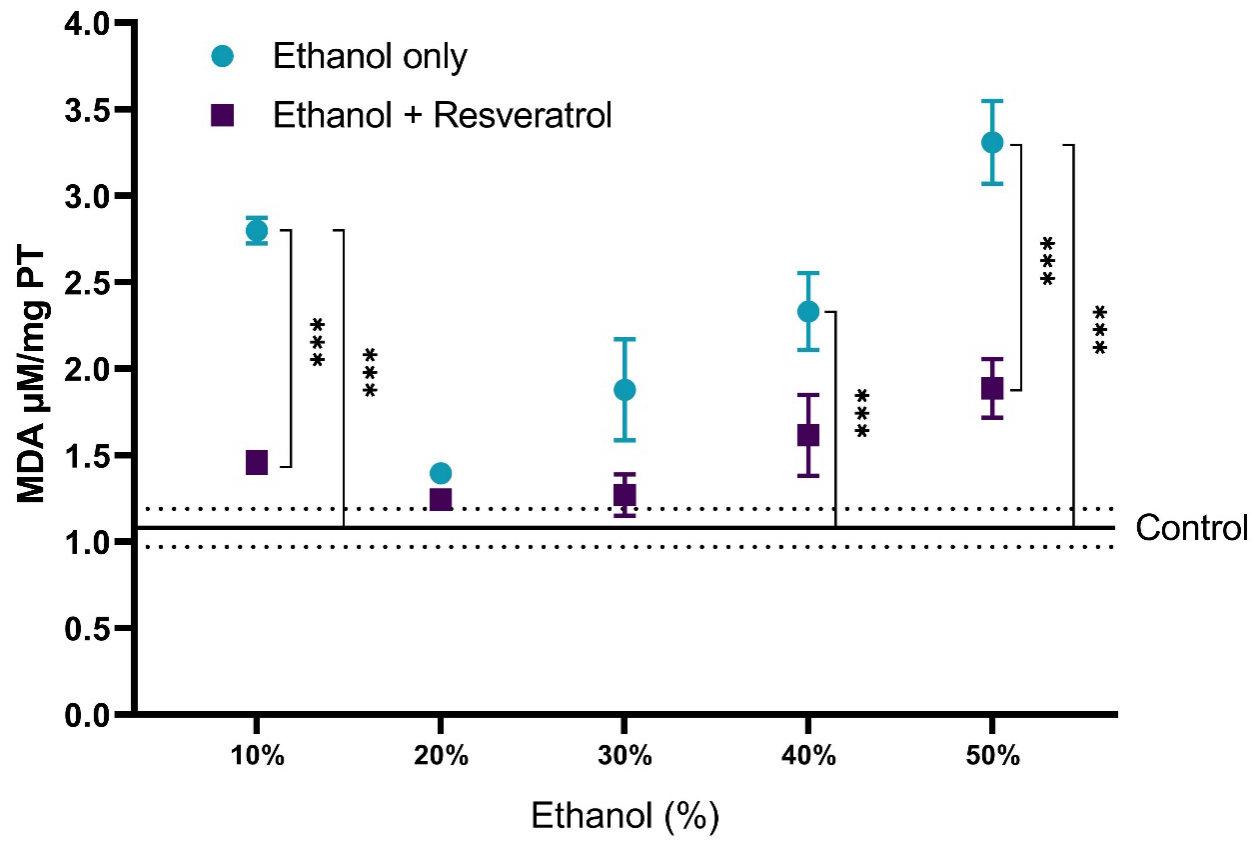
Antioxidant activity of resveratrol on MDA levels in the hippocampus of rats administered with ethanol and treated with resveratrol. Data are presented as mean ± SE. The straight line is the mean of the control group, and the dotted line is the SE. Comparison is made using a two-way fixed effects ANOVA model, and *post-hoc* comparison was made using Tukey’s test. *p < 0.05, **p < 0.01, ***p < 0.001.

**Figure 4:**
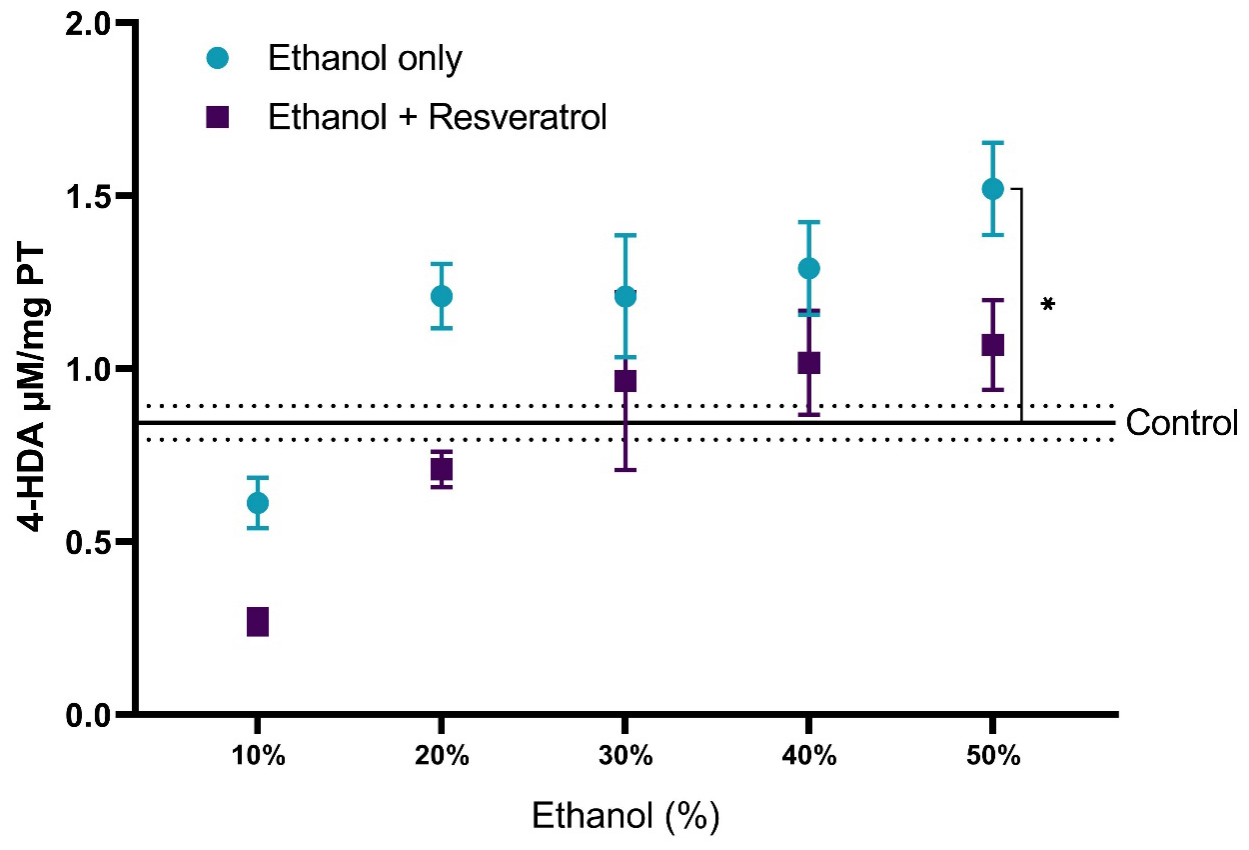
Antioxidant activity of resveratrol on 4-HDA levels in the hippocampus of rats administered. Data are presented as mean ± SE. The straight line is the mean of the control group, and the dotted line is the SE. Comparison is made using a two-way fixed effects ANOVA model, and *post-hoc* comparison was made using Tukey’s test. *p < 0.05, **p < 0.01, ***p < 0.001.

A comparison of each of the subproducts of lipid peroxidation was carried out; in Figure 3, we show the comparisons of the MDA values between the study groups, and in Figure 4, we show the results of the comparisons of the production of 4-HDA promoted by ethanol in different concentrations.

First, the mean MDA of the control group was 1.08 ± 0.11 μM/mg TP, the resveratrol group showed values similar to the control at all concentrations (all *p* > 0.05); meanwhile, the ethanol group showed statistically higher values at the concentrations at 10% (MD = –1.72, 95%CI: −2.53 to −0.91, *p* < 0.0001), 40% (MD = –1.25, 95%CI: −2.06 to −0.44, *p* = 0.0003), and 50% (MD = −2.23, 95%CI: −3.04 to −1.42, *p* < 0.0001). The ethanol group showed higher MDA values than the resveratrol group, but only in the concentrations at 10% (MD = 1.34, 95%CI: 0.53 to 2.15, *p* < 0.0001) and 50% (MD = 1.42, 95%CI: 0.61 to 2.23, *p* < 0.0001), statistically significant differences were observed. On the other hand, about the levels of 4-HDA, no statistical differences were observed between the study groups, and only at the concentration of 50%, the ethanol group had higher values than the control group (MD = -0.68, 95%CI: −1.28 to −0.07, *p* = 0.02).

### 3.3 Effect of resveratrol on catalase and SOD activity

**Figure 5:**
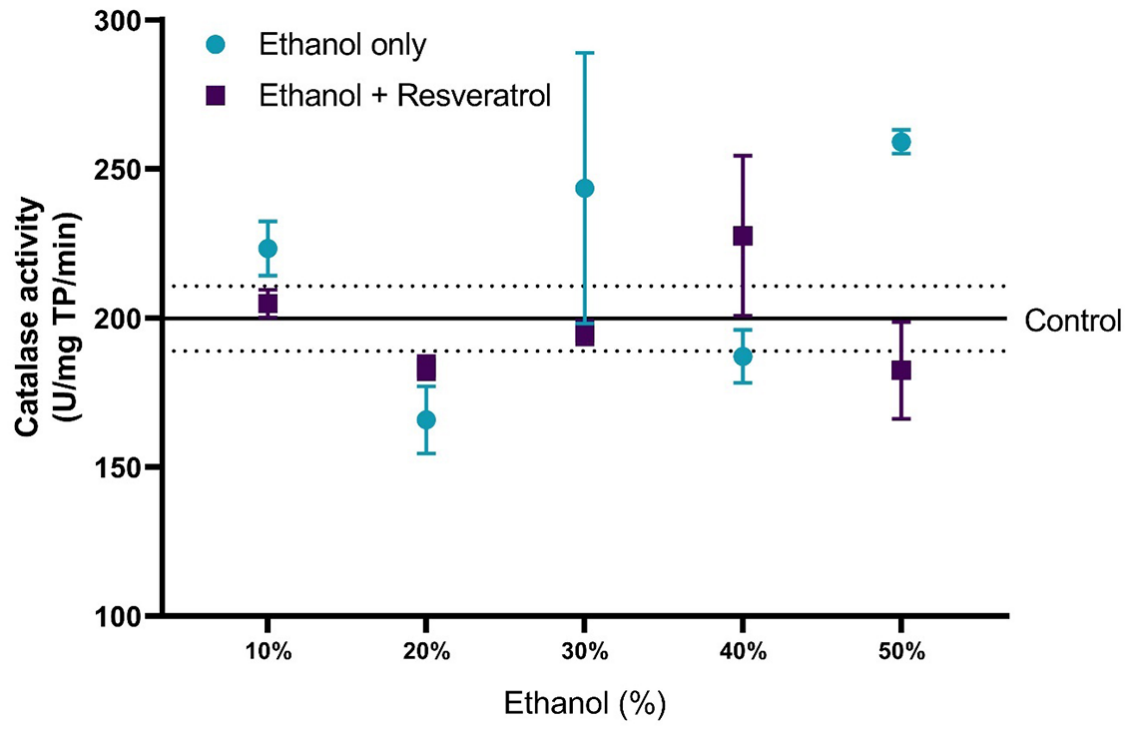
Antioxidant effect of resveratrol on catalase activity in the hippocampus of rats administered with ethanol. Data are presented as mean ± SE. The straight line is the mean of the control group, and the dotted line is the SE. Comparison is made using a two-way fixed effects ANOVA model, and *post-hoc* comparison was made using Tukey’s test. *p < 0.05, **p < 0.01, ***p < 0.001.

**Figure 6:**
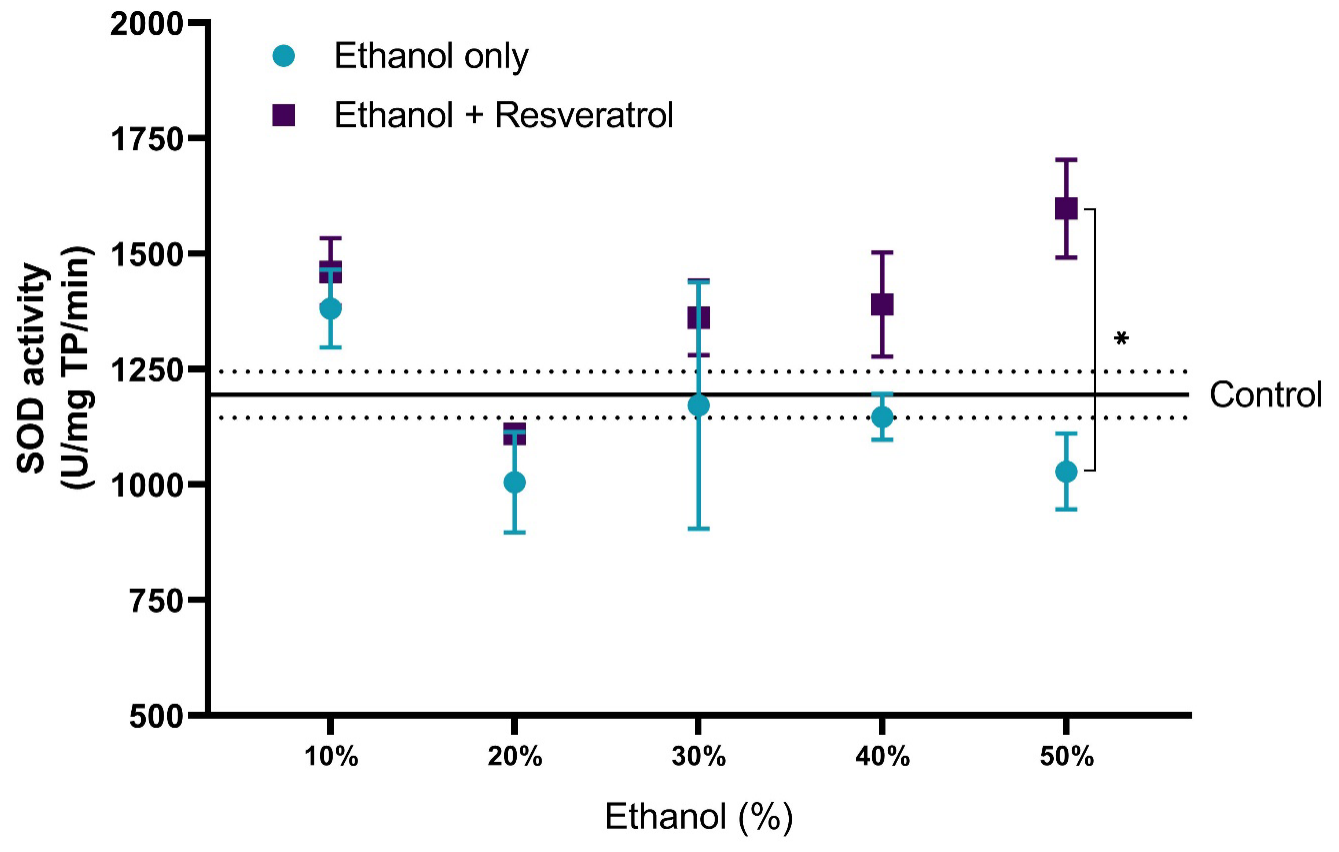
Antioxidant effect of resveratrol on superoxide dismutase activity on the hippocampus of rats administered ethanol. Data are presented as mean ± SE. The straight line is the mean of the control group, and the dotted line is the SE. Comparison is made using a two-way fixed effects ANOVA model, and *post-hoc* comparison was made using Tukey’s test. *p < 0.05, **p < 0.01, ***p < 0.001.

The comparisons of the antioxidant effect of the catalase between the study groups are shown in Figure 5. None of the ethanol concentrations administered significantly changed catalase activity when compared to the control group (199.80 ± 10.91 U/mg TP/min). Additionally, the animals treated with resveratrol showed no significant difference from the control or ethanol groups. In Figure 6, we show the results of the antioxidant effect of SOD between the study groups. The mean antioxidant effect in the control group was 1195.19 ± 50.07 U/mg TP/min. No significant differences were observed in any group, in any of the ethanol concentrations, and with respect to the control group (all *p* > 0.05), only a difference was observed between the study group at the 50% ethanol concentration (DM = –569.2, 95%CI: −1,087 to −51.62, *p* = 0.02).

### 3.4 Effect of resveratrol on the components of the glutathione system in the hippocampus

The results in the comparisons of the glutathione components between the study groups in the different concentrations of ethanol are shown in Table 1. The ethanol group showed differences with the control group only in Total-GSH at the 10% concentration and in GSH and GST at the 50% concentration. On the other hand, the resveratrol group showed statistically significant increases with respect to the control group Total-GSH and GSH, both at concentrations of 10–40%; in the case of GPx, the resveratrol group had statistically lower values than the control at all ethanol concentrations. For GST, resveratrol had higher values than the control group only at the 20 and 30% concentrations, while the values of GR were higher than the control from the 30% concentration onward. Between the ethanol and resveratrol groups, significant differences were observed at all ethanol concentrations in Total-GSH, GSH, and GPx. While in GST, differences were only observed in the concentrations of 20, 40, and 50%, and in GR, there were only differences in the highest concentrations (40 and 50%).

**Table 1:** Effects of resveratrol on the enzymatic activity of the glutathione system in the hippocampus of rats administered with ethanol.

	Control	Ethanol only	Ethanol + Resveratrol
**10% of ethanol**			
Total glutathione (Total-GSH)	1.2421 ± 0.0592	1.0144 ± 0.5036 ^a^	2.3967 ± 0.1770 ^a,b^
Reduced glutathione (GSH)	1.3727 ± 0.0754	1.5467 ± 0.0633	2.3967 ± 0.1770 ^a,b^
Oxidized glutathione (GSSG)	0.0145 ± 0.0010	0.0106 ± 0.0025	0.0104 ± 0.0003
Glutathione peroxidase (GPx)	1.8147 ± 0.0481	1.5533 ± 0.1567	1.5600 ± 0.0700 ^a,b^
Glutathione S-transferase (GST)	39.0080 ± 0.6760	39.6233 ± 3.0533	48.2533 ± 2.1967
Glutathione reductase (GR)	4.7587 ± 0.2162	3.2633 ± 0.5867	4.2933 ± 0.1967
**20% of ethanol**			
Total glutathione (Total-GSH)	1.2421 ± 0.0592	1.0554 ± 0.0209	2.1790 ± 0.1286 ^a,b^
Reduced glutathione (GSH)	1.3727 ± 0.0754	1.0667 ± 0.0033	2.1700 ± 0.1290 ^a,b^
Oxidized glutathione (GSSG)	0.0145 ± 0.0010	0.0112 ± 0.0024	0.0125 ± 0.0020
Glutathione peroxidase (GPx)	1.8147 ± 0.0481	1.7600 ± 0.1332	1.1067 ± 0.0120 ^a,b^
Glutathione S-transferase (GST)	39.0080 ± 0.6760	35.3933 ± 4.0709	54.5733 ± 1.3641 ^a,b^
Glutathione reductase (GR)	4.7587 ± 0.2162	4.9633 ± 0.3767	5.1733 ± 0.4667
**30% of ethanol**			
Total glutathione (Total-GSH)	1.2421 ± 0.0592	1.2434 ± 0.1595	2.1904 ± 0.1108 ^a,b^
Reduced glutathione (GSH)	1.3727 ± 0.0754	1.4107 ± 0.1293	2.1800 ± 0.1114 ^a,b^
Oxidized glutathione (GSSG)	0.0145 ± 0.0010	0.0128 ± 0.0027	0.0104 ± 0.0024
Glutathione peroxidase (GPx)	1.8147 ± 0.0481	1.9433 ± 0.0167	0.8133 ± 0.1468 ^a,b^
Glutathione S-transferase (GST)	39.0080 ± 0.6760	41.8700 ± 0.6213	51.1733 ± 2.4253 ^a^
Glutathione reductase (GR)	4.7587 ± 0.2162	5.6593 ± 0.1866	6.8167 ± 0.3031 ^a^
**40% of ethanol**			
Total glutathione (Total-GSH)	1.2421 ± 0.0592	1.0964 ± 0.1200	2.1754 ± 0.0545 ^a,b^
Reduced glutathione (GSH)	1.3727 ± 0.0754	1.2200 ± 0.0800	2.1667 ± 0.0536 ^a,b^
Oxidized glutathione (GSSG)	0.0145 ± 0.0010	0.0170 ± 0.0028	0.0087 ± 0.0009
Glutathione peroxidase (GPx)	1.8147 ± 0.0481	1.4767 ± 0.0633	1.0000 ± 0.0473 ^a,b^
Glutathione S-transferase (GST)	39.0080 ± 0.6760	29.5033 ± 1.4751	43.2067 ± 2.3710 ^b^
Glutathione reductase (GR)	4.7587 ± 0.2162	3.2367 ± 0.0667	7.7333 ± 0.3333 ^a,b^
**50% of ethanol**			
Total glutathione (Total-GSH)	1.2421 ± 0.0592	0.8800 ± 0.0569	1.8194 ± 0.0491 ^b^
Reduced glutathione (GSH)	1.3727 ± 0.0754	0.8500 ± 0.0839 ^a^	1.8167 ± 0.0491 ^b^
Oxidized glutathione (GSSG)	0.0145 ± 0.0010	0.0138 ± 0.0025	0.0069 ± 0.0009
Glutathione peroxidase (GPx)	1.8147 ± 0.0481	1.8100 ± 0.0577	0.8600 ± 0.0289 ^a,b^
Glutathione S-transferase (GST)	39.0080 ± 0.6760	22.7900 ± 1.1738 ^a^	40.8433 ± 1.6810 ^b^
Glutathione reductase (GR)	4.7587 ± 0.2162	3.3500 ± 0.2500	7.5867 ± 0.6220 ^a,b^

Data are presented as Mean ± standard error. Comparisons are made using the two-way fixed effects ANOVA model and a pairwise comparison using Tukey’s *post-hoc* test. a: statistically significant difference (*p* < 0.05) with respect to the control group; b: statistically significant difference (*p* < 0.05) with respect to the ethanol-only group.

### 3.5 Effect of resveratrol on locomotor behavior and memory

**Figure 7:**
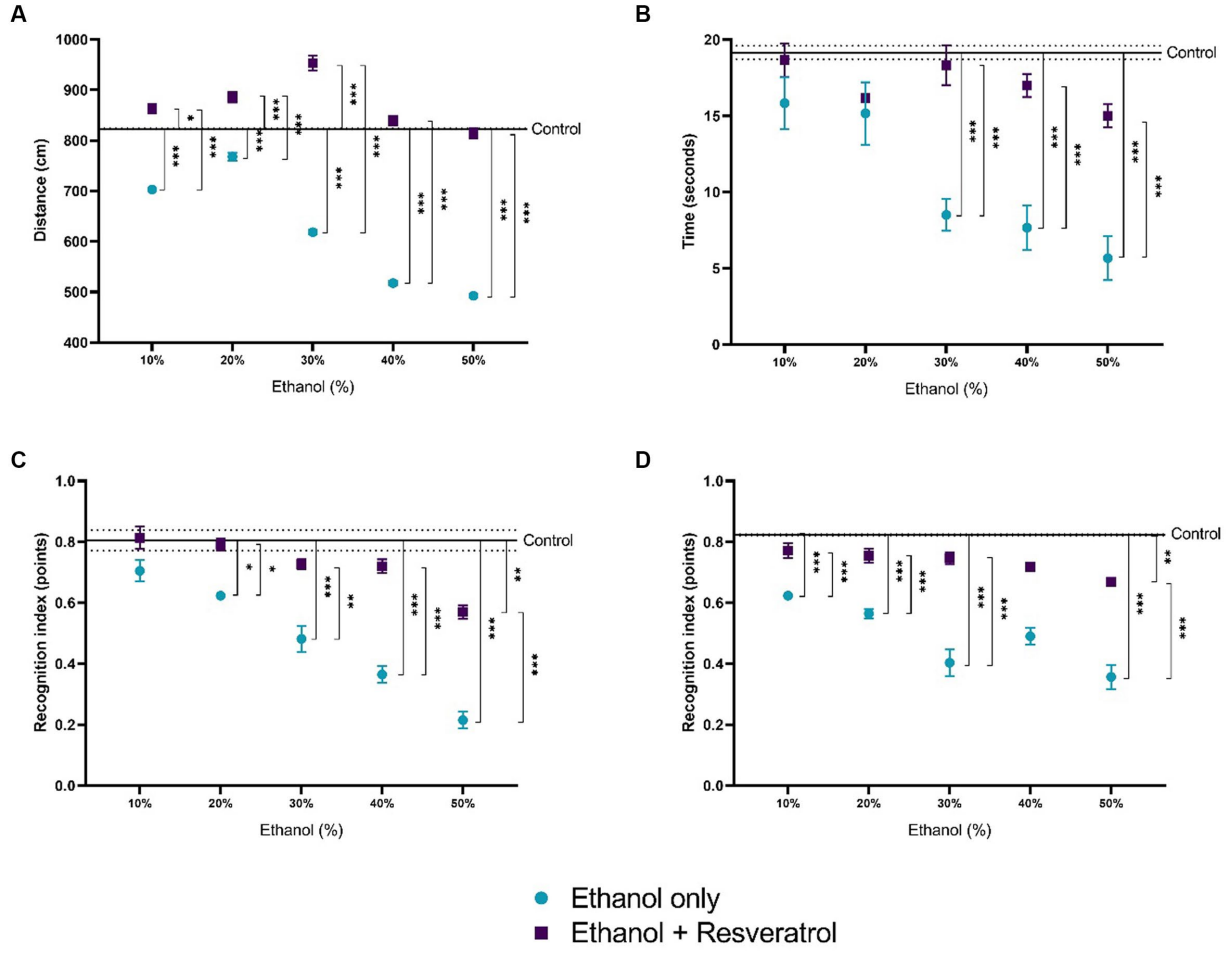
Effect of resveratrol on alterations in locomotor activity caused by ethanol. Data are presented as mean ± SE. **(A)** Distance traveled, **(B)** time in seconds of the exploration of two objects, **(C)** short-term recognition index, and **(D)** long-term recognition index. The straight line is the mean of the control group, and the dotted line is the SE. Comparison is made using a two-way fixed effects ANOVA model; *post-hoc* comparisons were made using Tukey’s test. *p < 0.05, **p < 0.01, ***p < 0.001.

The results of the four open field tests are shown in Figure 7. Figure 7A shows the comparison of the distance traveled between the study groups, and the mean distance traveled in the control group was 823.2 ± 1.01 cm. We observed that the distance traveled is lower at the highest concentrations of ethanol (30, 40, and 50%), being statistically significant for all concentrations (all *p* < 0.0001), in the resveratrol group, the distances do not differ from those of the control group in the concentrations of 40 and 50%, and in the lower concentrations (10, 20, and 30), the distance traveled in resveratrol group was greater than the control. Figure 7B shows the time in seconds of the exploration of two objects, the media time in the control group was 19.16 ± 0.44 s, and it was observed that the ethanol group at 30% concentration had shorter times than the control group and resveratrol group, but the resveratrol group showed no differences with the control group in any of the ethanol concentrations. Figure 7C shows the values of the short-term recognition index, the media in the control group was 0.81 ± 0.04 points, the ethanol group had lower index values compared to the control group and the resveratrol group at 20% concentration, while the resveratrol group only showed differences with the control at the 50% concentration. Figure 7D shows the values of the long-term recognition index, the media in the control group was 0.82 ± 0.002 points, and similar to the short-term, the ethanol group shows a lower score compared to the control and the resveratrol group, at 10, 20, 30, and 50% concentrations, while resveratrol group only showed differences with the control group at the 50% concentration.

## 4 Discussion

In this study, the effect of resveratrol on oxidative stress markers and endogenous antioxidant enzymes in the hippocampus of Wistar rats administered ethanol at 10, 20, 30, 40, and 50% concentrations was analyzed. It was observed that resveratrol had a protective effect for the elevation of nitrite production and lipid peroxidation products, mainly at high concentrations of ethanol, but it was not observed to have any specific effect on endogenous antioxidant enzymes. In various *in vivo* models, it has been shown that the continuous or periodic consumption of ethanol causes long-term brain problems in the adult brain by deregulating its redox balance, generating changes in various markers of oxidative stress such as NO and lipid peroxidation, that, depending on the pattern of consumption, the dose, and the period of exposure to ethanol, a variety of structural and functional brain deficits can be observed (Brocardo et al., 2016). At the end of the 1980s, a group of biomarkers for measuring oxidative stress was introduced, indicating the possible damage to macromolecules such as lipids and proteins, markers of physiological and cognitive functions, as well as the relationship with some specific pathology (Rossi et al., 2016), which are used with the aim of developing new preventive, diagnostic, and therapeutic strategies to prevent the appearance of various diseases (Grotto et al., 2009).

The groups given resveratrol show lower nitrite levels compared to those treated solely with ethanol. Prolonged administration of resveratrol over a 2-month period keeps, similar to the controls, the nitrite levels in the hippocampus of Wistar rats exposed to different ethanol concentrations ranging from 10 to 50%. This outcome aligns with the findings of Lorenz et al. (2003), who observed a notable reduction in nitrite levels in glial cells exposed to elevated ethanol concentrations following resveratrol treatment. Other studies carried out in recent years support the results found in this study, for example, Zima et al. (2001) showed that NO metabolites (nitrites and nitrates) increase in alcoholic patients (34.3, SD: 2.6 vs. 22.7, SD: 1.2 μmol/L); in addition, Ng and Lee (2019) found *in vitro* that high concentrations of NO diffuse into surrounding cells in the brain, which could be linked to increased cytotoxicity in neurons, glia, and myelin. Teixeira et al. (2014) found a significant reduction in the number of neurons and glial cells with an increase in nitrite levels in young rats administered ethanol. One of several possible mechanisms for alcohol-induced neurotoxicity may be attributable to the modulation of the hypothalamic–pituitary response to alcohol by nitric oxide generating systems. For example, alcohol can regulate NOS, a rate-limiting enzyme, so that NO increases its local levels, leading to neurotoxicity (Lancaster, 1995). It was recently shown that downregulation of NO synthesis by inhibition of nNOS enhanced the activity of ionotropic GABA receptors (GABAaRs), which favored greater permeability of chloride ion (Cl^−^) a higher concentration of Cl^−^ reduces neuronal excitability such as glutamatergic cells in the hippocampus and consequently a decrease in memory and learning processes (Colrain et al., 2014; Yu et al., 2019). While some articles provide backing for these theories, there is still a lack of a widely accepted mechanism to explain the neurotoxic effects of alcohol. However, the existing and previous evidence indicates that the immediate impact of alcohol consumption might trigger programmed cell death, leading to subsequent non-programmed degeneration of neurons as alcohol exposure increases (Seo and Rivier, 2003).

The results obtained in this study suggest that resveratrol could be beneficial for neurons since it prevented the accumulation of NO and its metabolites in all ethanol concentrations studied; the maintenance of NO observed in the study could avoid the neurotoxic effect, suggesting that resveratrol has the capacity to prevent the toxic effects of ethanol by maintaining nitrite levels, since these increase under conditions of high oxidative stress, in this case generated by the administration of ethanol in 30, 40, and 50% concentrations. On the other hand, part of the evidence of the possible protective effect of resveratrol was observed in behavioral evaluations, where it was found that in most of the tests, animals that received resveratrol together with ethanol had values similar to controls in open field tests. It is important to point out that NO has several physiological functions in the central nervous system; therefore, the evaluation of a single marker such as NO may be incorrect to dictate that there is oxidative stress, so in future studies, it should be studied in conjunction with other markers.

Lipoperoxidation is a complex process that involves the formation of different products with various biological functions such as regulation of gene expression and cell signaling (Forman et al., 2008). MDA and 4-HDA are generated after oxidation of biological membranes. These compounds are most called lipoperoxides and are used as markers of oxidative stress in plasma and tissues (Ayala et al., 2014). Hippocampal atrophy is one of the most prominent manifestations of alcoholism, often caused by the experimental administration of ethanol. Ramezani et al. (2012) found that chronic exposure to ethanol (35% orally ethanol solution) in rats significantly increased lipid peroxidation in the brain, as an indicator of oxidative stress in the cerebellum. Investigations carried out in the last decades maintain that the reduction of lipid peroxidation consequently allows for favoring the neuroprotective effect of resveratrol (Kasdallah-Grissa et al., 2006). The results of our study show that in the animals administered with ethanol at concentrations of 40 and 50%, the levels of MDA + 4-HDA increased, and an increase of 150.91% (SD: 8.17) was observed in the group administered with 50% ethanol compared to the control group; and although at lower concentrations (20 and 30%), higher ethanol levels were also observed than those in the control groups, there were no significant statistical differences. It is important to mention that these results cannot be taken as a dose effect of ethanol on the MDA + 4-HDA since the variable is categorical in nature and not a continuous quantitative variable with which a linear relationship can be made. The oral administration of 10 mg/kg/day of resveratrol maintained MDA + 4-HDA levels similar to those of the control in all ethanol concentrations, and this protective activity agrees with results previously obtained in the laboratory where it is observed that the administration of resveratrol with a 10% ethanol solution significantly decreases lipid peroxidation in the same tissue (Aguilar-Alonso et al., 2018).

*In vivo* and *in vitro* studies have reported that chronic ethanol administration significantly increases MDA levels (Grotto et al., 2009). Nahar and Jat (2021) demonstrated that chronic ethanol consumption caused a significant increase in MDA levels in rat brain synaptosomes. Similarly, Turkcu et al. (2010) found that MDA levels in rat brain tissue samples increased significantly in animals treated with ethanol compared to the control and that when an antioxidant was administered, these metabolites returned to their normal levels, acting as a protective agent. These findings are similar to those we observed in the hippocampus of rats used in this investigation, where we observed that MDA increased in the 40 and 50% concentrations of ethanol compared to the control group, but we do observe increases at lower concentrations; however, in the group of animals that were administered resveratrol plus ethanol, the MDA levels remained similar to the levels observed in the control group at all ethanol concentrations, and this could be interpreted as the protective effect of resveratrol that is mentioned in the aforementioned studies.

A study conducted by Petrovic et al. (2020) demonstrated that the marked increase in the product of lipid peroxidation (4-HDA) and the decrease in specific neuronal neurofilaments support the idea that the peroxidation process and the disruption of the cytoskeleton neurodegeneration could be initial steps in alcohol-associated neurodegeneration. The dual role of 4-HDA is known, and high concentrations are extremely toxic, leading to cell death. Prior to this event, many effects occur, such as rapid glutathione depletion, protein damage, increased lipid peroxidation, impaired calcium homeostasis, inhibition of DNA, RNA, and protein synthesis, inhibition of respiration and glycolysis, lactate release, and morphological changes (Esterbauer et al., 1991). It is known that 4-HDA is produced in less quantity compared to MDA during the lipid peroxidation process; in this investigation, we observed that both the animals in the ethanol-only group and the ethanol + resveratrol group had 4-HDA levels similar to those of the control group, and differences were only observed in the highest ethanol concentration (50%).

Regarding catalase, the administration of any concentration of ethanol did not show significant changes, and in the 50% concentration of ethanol, the enzymatic activity had an increase of 30.48% (SD: 12.65) compared to the control group, without presenting a statistical difference, while with the administration of resveratrol showed a less catalase enzymatic activity of 29.64% (SD: 10.33) compared to the ethanol group in the 50% concentration, but also, without a statistical difference. It seems that at these concentrations, this enzyme does not need to be activated or increase its activity; however, with the administration of 50% ethanol, a significant increase is observed in various investigations that have shown more catalase activity, and the deficient activity of the endogenous enzymatic system leads to a greater production of free radicals and consequently greater cell damage (Ledesma et al., 2013).

Regarding the effect of resveratrol on antioxidant enzyme activity, SOD did not show statistically significant changes in rats administered with any concentration of ethanol, maintaining similar levels to the control group; on the other hand, the group treated with resveratrol showed an increase in enzymatic activity in the doses of 40 and 50% of 21.24% (SD: 13.86) and 56.9% (SD: 25.6), respectively, with respect to the group administered with ethanol; however, only in the 50% concentration, a statistically significant difference was observed. It is important to mention that although in the groups administered with 40 and 50% ethanol, no significant difference was observed in the activity of this enzyme with respect to the control group, when using resveratrol, its activity did increase, showing a significant difference with respect to the ethanol group up to a concentration of 50%. This may be because, at concentrations lower than 50% ethanol, resveratrol acts as a scavenger, eliminating excess free radicals; however, at higher concentrations, it is not capable of reducing stress levels and begins to act as an inducer of the enzymatic activity.

The glutathione antioxidant system includes enzymatic and non-enzymatic elements. Glutathione represents the predominant source of antioxidants in the central nervous system and plays a crucial role in preserving redox homeostasis (Aoyama, 2021). It is important to mention that the behavior of this system varies in different brain regions. When the concentration of free radicals [reactive oxygen species (ROS) and reactive nitrogen species (RNS)] is very high, the endogenous antioxidant system composed of enzymes such as catalase, SOD, and the enzymes of the glutathione system is activated to reduce said concentration. Specifically, when numerous free radicals are within the cell, GPx becomes active, diminishing the reactive species and converting GSH into its oxidized form (GSSG). Consequently, GSSG undergoes reduction to GSH through the enzymatic action of GR, thereby reinstating the initial GSH levels. Throughout this mechanism, there is a continuous turnover of GSH and its oxidized counterpart, GSSG, as substrates for the enzymes involved. Our results show that in the different concentrations of ethanol used, GR increases significantly in the groups administered with resveratrol but not GPx; they are even minor, which shows the very low concentration of glutathione in its oxidized form. Sommavilla et al. (2012) found that after chronic administration with ethanol, GPx activity was elevated, which is consistent with our groups administered only with ethanol. Therefore, it is demonstrated that resveratrol allows glutathione to be kept active and in adequate concentrations in its reduced form, even under attack conditions such as higher concentrations of ethanol. GST is another antioxidant detoxification enzyme that participates in the detoxification of oxidized products by conjugating GSH with electrophilic centers on many toxic substrates to form non-toxic products (Mazari et al., 2023). In our results, this enzyme appears active in the animals administered with resveratrol but without showing differences between the control group and the group treated only with ethanol. This shows the strengthening of the endogenous antioxidant system caused by resveratrol.

It is well known that ethanol is a molecule capable of crossing up to 90% of the blood–brain barrier; this is one of the reasons why it is attributed to a direct effect. Various studies have shown that ethanol can interfere with nerve action potentials and influence behavior and short- and long-term recognition memory (Baliño et al., 2021). When ethanol is ingested, biochemical changes occur until it is converted into acetaldehyde, the latter passes into the blood and produces intoxication, which is largely caused by its accumulation and an earlier generation of ROS. In the brain, ethanol favors synaptic inhibition produced by the GABA transmitter; the anesthetic effect is carried out mainly through an inhibitory action on the NMDA receptors of the neurotransmitter glutamate, which has an excitatory power in the brain (Pascual et al., 2011) producing modifications in the neuronal mechanism of the hippocampus and cortex, which could constitute neurochemical correlates of memory loss, and this would also explain why the animals that were administered with high concentrations of ethanol their locomotor activity decreases. On the other hand, it is known that acute or chronic exposure to ethanol must be metabolized by dehydrogenases and by the microsomal ethanol oxidation system that is related to the production of NADH, NADP + and in greater quantities ROS (Jiang et al., 2020). Subsequently, ROS levels and the resulting oxidative stress represent an important threat to cell survival since macromolecules such as polyunsaturated fatty acids in cell membranes are altered, protein misfolding occurs, and there is a decrease in the endogenous defense system, which in turn leads to the malfunction of neuronal cells. These oxidative damages accumulate until finally changes in neurochemistry and neuronal plasticity are reflected, thus causing behavioral problems. Resveratrol is an antioxidant par excellence; given its chemical structure, it can greatly reduce the negative effects caused by ROS by acting as a scavenger and stabilizing free radicals before they cause cellular damage.

The reason why a single dose of resveratrol (10 mg/Kg/day) is used in our study is that, to the best of our knowledge, there is no conclusive evidence of what would be the best dose of resveratrol for health benefits; the more recent studies in animal models show that both low (<20 mg/Kg/day) and high (>30 mg/Kg/day) doses of resveratrol have similar effect sizes on clinical outcomes such as ischemia/reperfusion injury (Xue et al., 2022) and pulmonary arterial hypertension (Ferreira et al., 2020). Unfortunately, at the date of writing this study, there is no meta-analytic evidence on the most useful dose for the protection of oxidative stress, so we consider it widely useful to evaluate a single low dose to reduce the confounding factor of dose-dependence. In turn, it could serve as evidence, for developing countries (such as Mexico) of the usefulness of low doses of resveratrol that imply lower costs in the translation to supplementation in humans. We would like to add that the use of different concentrations of ethanol in this study tries to simulate the different types of consumption of different beverages that exist in the consumer market for human beings. The 10% ethanol concentrations were used to simulate the consumption of natural or unfortified wines that contain between 8 and 12% ethanol, although some varieties have a somewhat higher content, ranging between 12 and 14%; therefore, for the latter, the concentration of 20% was used. The concentrations of 30, 40, and 50% try to simulate alcohol concentration in brandy that ranges between 35 and 60% depending on how it is prepared and also for spirits, including vodka, rum, and whiskey, which typically contain between 30 and 50% alcohol. A standard drink contains between 0.5 and 0.7 fluid ounces of absolute alcohol (one ounce is approximately 30 mL.). Therefore, a 1.5-ounce (45 mL) shot of vodka, a 5-ounce (150 mL) glass of wine, and a 12-ounce (355 mL) bottle of beer induce the same effects.

The present study has several limitations that must be taken into account while reading. The first is that there was no positive control group in which only resveratrol was given, and the absence of this positive control may bias the comparison to not have an estimated effect of resveratrol outside of ethanol concentrations. The second limitation is that it cannot be considered with 100% reliability that the oxidative stress effects of ethanol are only due to its consumption since the experimental animals were subjected to stress by being forced to consume ethanol by administrations and not by free access consumption. The third limitation of our study is that a single dose of resveratrol was used for the five study groups of ethanol + resveratrol. This was done since it was intended to evaluate the effect of said single dose in various concentrations, but when using a single dose, the study of the dose effect of resveratrol on oxidative stress markers is impossible. The fourth limitation of our study is that we cannot demonstrate that resveratrol reduces oxidative stress markers since it was administered together with ethanol in the same study period. Our study only shows that resveratrol maintains similar levels to the control group in various markers of oxidative stress or antioxidant enzymes. The fifth limitation of our study is that no data on plasma ethanol concentrations were obtained for any of the study groups; this would be important to facilitate the understanding of the effects of each ethanol concentration used.

## 5 Conclusion

The administration of high concentrations of ethanol for a period of 2 months in Wistar rats gradually causes an increase in NO metabolites (nitrites), products of the lipid peroxidation process (MDA and 4-HDA), but not the modification of the activity of catalase and SOD enzymes; by contrast, the administration of resveratrol in a single dose of 10 mg/kg/day promotes protection against the oxidative stress generated by high concentration of ethanol. Under the conditions of the present investigation, resveratrol did not modify the enzyme activity of superoxide dismutase and catalase in administrations of less than 50% ethanol but allowed glutathione to be kept active and in adequate concentrations in its reduced form and avoided alterations in the locomotor system.

## Data availability statement

The original contributions presented in the study are included in the article/supplementary material, further inquiries can be directed to the corresponding author.

## Ethics statement

The animal study was approved by Institutional Committee for the Care and Use of Laboratory Animals with project folio 100426322-CICUALVIEP-18/3. The study was conducted in accordance with the Mexican legislation and institutional requirements.

## Author contributions

AN-C: Conceptualization, Writing – original draft. DJ-S: Investigation, Validation, Writing – review & editing. IC-A: Investigation, Resources, Writing – review & editing. AK-G: Data curation, Formal analysis, Investigation, Writing – original draft. JG-D: Writing – review & editing. OV-L: Visualization, Writing – review & editing. ML-H: Formal analysis, Visualization, Writing – review & editing. IP-X: Visualization, Writing – review & editing. OS-B: Funding acquisition, Methodology, Resources, Writing – original draft.
